# Multiomics characterization and verification of clear cell renal cell carcinoma molecular subtypes to guide precise chemotherapy and immunotherapy

**DOI:** 10.1002/imt2.147

**Published:** 2023-11-16

**Authors:** Jialin Meng, Aimin Jiang, Xiaofan Lu, Di Gu, Qintao Ge, Suwen Bai, Yundong Zhou, Jun Zhou, Zongyao Hao, Fangrong Yan, Linhui Wang, Haitao Wang, Juan Du, Chaozhao Liang

**Affiliations:** ^1^ Department of Urology The First Affiliated Hospital of Anhui Medical University, Institute of Urology, Anhui Medical University, Anhui Province Key Laboratory of Genitourinary Diseases Anhui Medical University Hefei China; ^2^ Department of Urology, Changhai Hospital Naval Medical University (Second Military Medical University) Shanghai China; ^3^ Department of Cancer and Functional Genomics Institute of Genetics and Molecular and Cellular Biology, CNRS/INSERM/UNISTRA Illkirch France; ^4^ The Second Affiliated Hospital, School of Medicine The Chinese University of Hong Kong, Shenzhen & Longgang District People's Hospital of Shenzhen Shenzhen China; ^5^ Department of Surgery, Ningbo Medical Center Lihuili Hospital Ningbo University Ningbo Zhejiang China; ^6^ Research Center of Biostatistics and Computational Pharmacy China Pharmaceutical University Nanjing China; ^7^ Cancer Center, Faculty of Health Sciences University of Macau Macau SAR China; ^8^ Present address: Center for Cancer Research Bethesda Maryland USA

**Keywords:** clear cell renal cell carcinoma, immune activation, molecular subtyping, multiomics

## Abstract

Clear cell renal cell carcinoma (ccRCC) is a heterogeneous tumor with different genetic and molecular alterations. Schemes for ccRCC classification system based on multiomics are urgent, to promote further biological insights. Two hundred and fifty‐five ccRCC patients with paired data of clinical information, transcriptome expression profiles, copy number alterations, DNA methylation, and somatic mutations were collected for identification. Bioinformatic analyses were performed based on our team's recently developed R package “MOVICS.” With 10 state‐of‐the‐art algorithms, we identified the multiomics subtypes (MoSs) for ccRCC patients. MoS1 is an immune exhausted subtype, presented the poorest prognosis, and might be caused by an exhausted immune microenvironment, activated hypoxia features, but can benefit from PI3K/AKT inhibitors. MoS2 is an immune “cold” subtype, which represented more mutation of VHL and PBRM1, favorable prognosis, and is more suitable for sunitinib therapy. MoS3 is the immune “hot” subtype, and can benefit from the anti‐PD‐1 immunotherapy. We successfully verified the different molecular features of the three MoSs in external cohorts GSE22541, GSE40435, and GSE53573. Patients that received Nivolumab therapy helped us to confirm that MoS3 is suitable for anti‐PD‐1 therapy. E‐MTAB‐3267 cohort also supported the fact that MoS2 patients can respond more to sunitinib treatment. We also confirm that SETD2 is a tumor suppressor in ccRCC, along with the decreased SETD2 protein level in advanced tumor stage, and knock‐down of SETD2 leads to the promotion of cell proliferation, migration, and invasion. In summary, we provide novel insights into ccRCC molecular subtypes based on robust clustering algorithms via multiomics data, and encourage future precise treatment of ccRCC patients.

## Highlights

- Three molecular subtypes of ccRCC based on multiomics data were identified, with diverse overall survival time, and validated in external cohorts.
- MoS1 is an immune exhausted subtype, which can benefit from PI3K/AKT inhibitors; MoS2 is an immune “cold” subtype, but is more suitable for sunitinib therapy; MoS3 is an immune “hot” subtype, which can benefit from anti‐PD‐1 immunotherapy.
- SETD2 is a tumor suppressor in ccRCC, and knock‐down of SETD2 leads to the promotion of cell proliferation, migration, and invasion.

## Graphical abstract

We established a novel molecular classification for ccRCC via multiomics features, named multiomics subtype (MoS). MoS correlates with significantly different clinicopathological parameters, molecular alterations, signaling activation, immunocyte infiltration, and therapeutic response. MoS1 subtype, presented the poorest prognosis, and might be caused by an exhausted immune microenvironment, activated hypoxia features, but can benefit from PI3K/AKT inhibitors. MoS2 subtype represented more mutations of VHL and PBRM1, favorable prognosis and is more suitable for sunitinib therapy. MoS3 is the immune “hot” subtype, and can benefit from the anti‐PD‐1 immunotherapy. We also confirm that SETD2 is a tumor suppressor in ccRCC, along with the decreased SETD2 protein level in advanced tumor stage, and knock‐down of SETD2 leads to the promotion of cell proliferation, migration, and invasion.

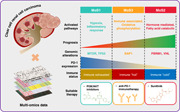

## INTRODUCTION

Kidney cancer is the ninth most common cancer among all tumor types in a global context [1]. Renal cell carcinoma (RCC) accounts for 90% of such malignancies [2]. With 426,800 male and 22,660 female onsets diagnosed in 2018, RCC was reported as the sixth and tenth most common tumors in American males and females, respectively [3]. The incidence rates of RCC varied significantly worldwide. Higher incidence rates were observed in North America compared to that in Asia and South America. It was also dissimilar across different regions, even within a county. For instance, 3.6 would develop RCC per 100,000 persons in Salerno, while 9.0 per 100,000 in the North East region of Italy [4]. Evidence has also shown that the incidence rate would increase with age, and the elderly more than 75 is the peak of morbidity [5]. According to the Surveillance, Epidemiology, and End Results Program, the incidence rates showed significant difference from race to race as well as genders. The incidence rates were highest among African Americans, while the rates for Asian/Pacific Islanders were only half that of other ethnic groups. Regardless of countries and races, incidence rates in males were twice as high as females at the same age levels [6]. For each individual, there are still many risk factors of RCC that have been validated, such as smoking, hypertension, and obesity [7, 8, 9, 10]. The overall incidence is increasing under the impact above in recent decades. However, the relative survival rates improved, for which the early detection with the application of abdominal imaging clinically and the comprehensive treatment strategies [11].

At present, RCC was divided into nine subgroups based on multiple histopathological features, such as anatomical locations, predominant cytoplasmic and staining features, structural and morphological features. Clear cell renal cell carcinoma (ccRCC) is the major subtype in the WHO classification, which accounts for 65%–70% of all subtypes of RCC [12]. ccRCC is a heterogeneous tumor with different genetic and molecular alterations [13]. Despite the overall survival (OS) rates of RCC are increasing, ccRCC is still one of the most malignancy urinary tumors causing 90,000 mortalities annually [14, 15]. Considering the high proportion in RCC and high mortality, a predictive instrument and an efficient classification are urgently needed. Currently accepted prognostic tools are based on the TNM stage and the histological differentiation grade [16, 17]. Nevertheless, the results are determined on abdominal imaging and clinicopathology, which may lead to an approximate margin of error of 20% [18, 19]. Therefore, the clinical values of these tools are limited. Further studies on a more efficient and accurate approach should be conducted. Fortunately, the development of microarray and high‐through sequencing technology offer an opportunity to subtype the ccRCC at molecular level, which may contribute to more precise guidance clinically [20]. Several research have been reported on this field based on single messenger RNA (mRNA) expression or protein features, which have advanced the understanding of crosstalk between ccRCC molecular characteristics and biological behaviors [20, 21, 22, 23, 24, 25]. However, none of these studies at single genomics level thoroughly unveiled the complicate links of molecular features and clinical characteristics in ccRCC. Thus, schemes for ccRCC classification system based on multi‐omics were proposed, which could promote further biological insights on ccRCC.

Large‐scale efforts were made to gather genomic profiles, transcriptomic profiles, and epigenetics profiles of ccRCC by The Cancer Genome Atlas (TCGA). Furthermore, many molecular alterations and their corresponding biological process have been revealed. These evidence provide fundamentals to comprehensively delve the classification of ccRCC. There have been several proposed multi‐omics integration strategies and classifiers of ccRCC. Song et al. found FOXM1 could act as a novel prognostic biomarker of ccRCC and provide a basis for early diagnosis at molecular level [26]. An increasing number of studies emphasize the intricate interactions within various omics of tumors. This analytical approach, which amalgamates findings from multiple omics, enables a comprehensive elucidation of tumor molecular subtypes, prognosis, and drug sensitivity [27, 28, 29]. In our study, a consensus ensemble was performed from different subtypes confirmed by multiple clustering algorithms, which included mRNA and long noncoding RNA (lncRNA) expression, somatic genetic mutation, copy number alteration (CNA), and DNA methylation profiles. Finally, a novel integrative consensus classification was established based on the consensus ensemble. It provided more comprehensive and further understanding of the correlation between molecular features and biological behaviors, survival prediction, and estimate of therapeutic effect.

## RESULTS

### Identify three molecular subtypes based on the TCGA‐KIRC cohort

**Figure 1:**
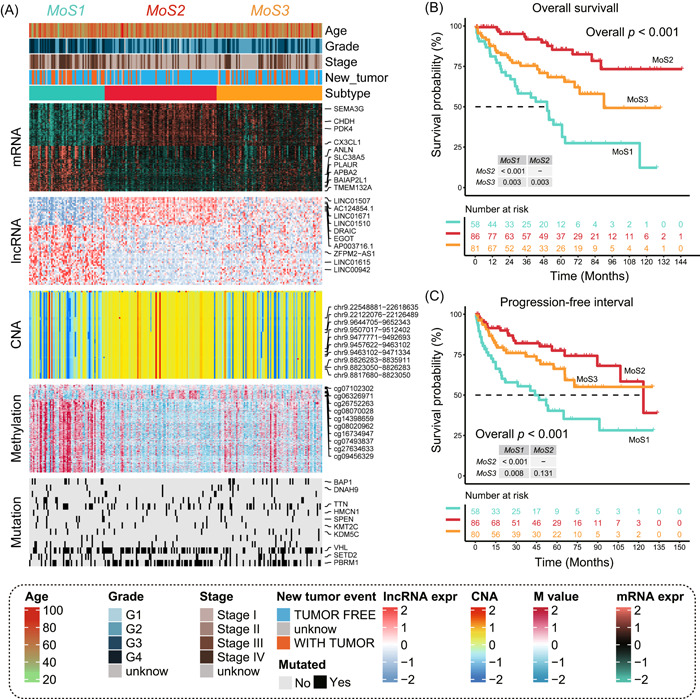
Identification of three distinct MoS clusters and evaluation of diverse clinical outcome. (A) Multiomics features of the MoS1, MoS2, and MoS3 subtypes. (B) Kaplan–Meier plot of overall survival for the three subtypes. (C) Kaplan–Meier curve of progression‐free interval for the three subtypes. CNA, copy number alteration; M value, methylation value; MoS, multiomics subtype.

After filtering the patients with all the data of mRNA, lncRNA expression, CNAs, DNA methylation, gene mutation, and OS outcome, 225 ccRCC patients from TCGA‐KIRC cohort was enrolled for the consensus analysis of molecular subtypes. With the recommendation number of multiomics clusters from clustering prediction index (CPI) and Gap statistics, we finally chose the number of 3 to identify the molecular differences (Figure S1A). Ten traditional algorithms blocking in the R package “MOVICS” were used to separate the patients to the pre‐set three multiomics subtypes (MoSs), and finally integrated into a robust classification via the ensemble consensus. The results from the silhouette analysis also illustrate the moderate similarity of samples in each cluster, with silhouette score of 0.75, 0.50, and 0.28 to MoS1, MoS2, and MoS3, respectively (Figure S1B). The distribution of above‐mentioned multi‐omics data in three MoSs displayed in Figure 1A, as well as the clinicopathological features. MoS2 contained more CNA, but less DNA methylation, patients with mutation of *VHL* and *PBRM1* were also separated to MoS2. The mRNA level of *SEMA3G, CHDH, PDK4* is highest in MoS2, while MoS1 contained more DNA methylation and high level of *ANLN, APBA2*, and *TMEM132A*. We also observed that more mutation of *VHL, SETD2*, and *PBRM1* in MoS3 group (Figure 1A).

### Patients with different subtypes faced diverse clinical outcomes

Clinical prognosis outcomes for tumor holders are very important to choose the radical or passive treatment, and letting patients have an expectation for future life. We revealed that more patients in MoS1 obtained advanced G4 tumor stage than any other MoSs (MoS1, 41.4% vs. MoS2, 4.7% vs. MoS3, 11.1%, *p* < 0.001, Table 1), and Stage IV (MoS1, 29.3% vs. MoS2, 7.0% vs. MoS3, 17.3%, *p* < 0.001, Table 1). Meanwhile, the average age of patients in three MoSs showed no difference (MoS1, 61.64 ± 11.93 vs. MoS2, 60.99 ± 11.14, vs. MoS3, 60.49 ± 11.44, *p* = 0.852, Table 1). We also observed the contrastive prognosis in three MoS subtypes, MoS2 had the better OS, and MoS1 contained the poor prognosis, the average OS time decreased from MoS2, MoS3 to MoS1 (MoS1, 37.74 ± 30.42 vs. MoS2, 56.39 ± 37.15, vs. MoS3, 44.84 ± 33.45 months, *p* < 0.001, Figure 1B), as well as the progression‐free interval time (MoS1, 31.49 ± 31.79 vs. MoS2, 47.25 ± 34.44, vs. MoS3, 38.74 ± 31.67 months, *p* < 0.001, Figure 1C).

**Table 1:** Summarization of the clinical parameters in subtypes.

Parameters	MoS1	MoS2	MoS3	*p* Value
Numbers	58	86	81	
Death events (%)	32 (55.2)	11 (12.8)	24 (29.6)	<0.001*
Age	61.64 ± 11.93	60.99 ± 11.14	60.49 ± 11.44	0.852
Grade (%)				
G1	0 (0.0)	4 (4.7)	0 (0.0)	<0.001*
G2	12 (20.7)	39 (45.3)	39 (48.1)	
G3	21 (36.2)	37 (43.0)	33 (40.7)	
G4	24 (41.4)	4 (4.7)	9 (11.1)	
Unknown	1 (1.7)	2 (2.3)	0 (0.0)	
Stage (%)				
Stage I	15 (25.9)	55 (64.0)	42 (51.9)	<0.001*
Stage II	6 (10.3)	11 (12.8)	7 (8.6)	
Stage III	20 (34.5)	14 (16.3)	16 (19.8)	
Stage IV	17 (29.3)	6 (7.0)	14 (17.3)	
Unknown	0 (0.0)	0 (0.0)	2 (2.5)	
New tumor (%)				
Tumor free	27 (46.6)	70 (81.4)	53 (65.4)	<0.001*
With tumor	27 (46.6)	11 (12.8)	25 (30.9)	
Unknown	4 (6.9)	5 (5.8)	3 (3.7)	

* *p* < 0.05.

### Silence of hypoxia signaling in MoS2 resulted the favorable prognosis

To further understand the difference of three subtypes, we conducted the Gene Ontology (GO) terms pathway enrichment to identify subtype‐specific activated signaling pathways. We observed that MoS1 patients contained the activation of membrane protein targeting, collagen metabolism, and acute inflammatory response (Figure S2A,B). As for MoS2, endothelium development, fatty acid metabolic and catabolic, mRNA processing pathways were enriched (Figure S2A,C). Meanwhile, the activation of immune‐associated pathways enriched in MoS3 significantly, including macrophage migration, antigen processing and presentation, regulation of cytokine production, and immune response regulation cell surface receptor signaling (Figure S2A,D). Hypoxia was reported tightly associated with the tumorigenesis of ccRCC [47, 48]. Therefore, we assessed the hypoxia status in three subtypes of ccRCC patients, from Winter hypoxia score [49], Buffa hypoxia score [50], and Ragnum hypoxia score [51], we all observed the lowest hypoxia scores in MoS2, which represent the inactivated of hypoxia signaling, consistent with the favorable prognosis of MoS2 (Figure S2E).

### Activated immune microenvironment presented in MoS3 and linked with immunotherapy response

**Figure 2:**
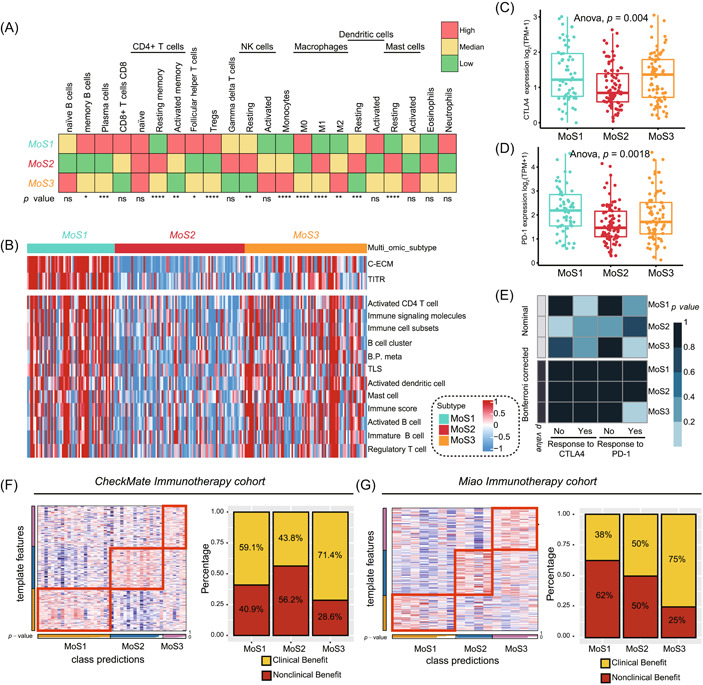
Differential immune microenvironment and immunotherapeutic response among three subtypes. (A) Immunocytes infiltration landscape of in MoSs. (B) Comparison of the immune microenvironment activated and exhausted status along with the signatures between MoS1, MoS2, and MoS3 subtypes. (C) Comparison of the expression level of CTLA4. (D) Comparison of the expression level of PD‐1. (E) SubMap showing the different response to anti‐PD‐1 or anti‐CTLA‐4 therapy within three MoSs. (F) Evaluation of the response to Nivolumab of MoS1, MoS2, and MoS3 subtypes based on CheckMate immunotherapy cohort. (G) Evaluation of the response to Nivolumab of MoS1, MoS2, and MoS3 subtypes. C‐ECM, cancer‐associated extracellular matrix; TITR, tumor‐infiltrating T regulatory cell; TLS, tertiary lymphoid structures.

We compared the immunocytes infiltration in MoSs by CIBERSORT, MoS1 contained the most infiltration of memory B cells, plasma cells, CD8+ T cells, CD4+ T cells, and M0 macrophages. MoS2 contained the proinflammatory immunocytes of M1 macrophages, gamma delta T cells, and eosinophils, which might be another reason for the favorable prognosis of MoS2. As for the MoS3 subtype, we observed the highly infiltrated monocytes and anti‐inflammatory M2 macrophages (Figure 2A). Furthermore, we compared the immune microenvironment activated status along with the signatures prior published [40], we found that both MoS1 and MoS3 contained the high infiltration of immunocytes, but MoS1 also contained the dramatical activation of tumor‐infiltrating T regulatory cell (TITR) and cancer‐associated extracellular matrix (C‐ECM) signatures, which represented the exhausted immune status (Figure 2B). As for the mRNA level of immune checkpoints, we revealed that MoS3 contained the higher CTLA4 level than MoS1 and MoS2 (*p* = 0.004, Figure 2C), while MoS1 contained the highest PD‐1 level than the other two subtypes (*p* = 0.0018, Figure 2D). We predicted the potential response to anti‐CTLA4 and PD‐1 therapy as compared with the melanoma treatment responders by Submap, the result showed that patients in MoS3 can benefit from the immunotherapy of anti‐PD‐1, but not MoS1 and MoS2 (Figure 2E). In grouping the samples in the external cohort, the top 300 specific maker genes were selected by the NTP analysis (Table S1). To validate the new findings that MoS3 are more appropriate for immunotherapy, we collected the mRNA expression profile and response results of anti‐PD‐1 therapy from CheckMate cohort and Miao cohort (Table S2). Patients from CheckMate cohort was firstly separated to three MoSs and 71.4% patients in MoS3 met the clinical benefit, higher than the rate in MoS1 (59.1%) and MoS2 (43.8%) (Figure 2F). As for Miao cohort, we observed similar result that 80% patients belong to MoS3 met the clinical benefit, but the rate lower to 38% in MoS1, 50% in MoS2 (Figure 2G).

### SETD2 mutation promoted poor prognosis of ccRCC patients via PLXNA2

**Figure 3:**
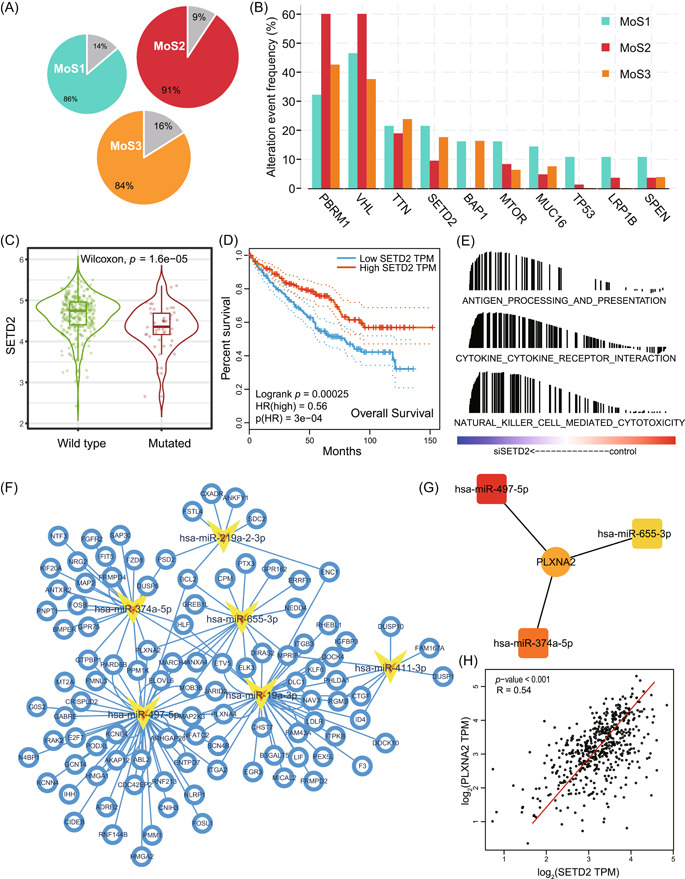
Elucidation of mutated genes among three subtypes and the crucial role SETD2 in ccRCC. (A) The proportion of patients with gene mutations within MoS1, MoS2, and MoS3 subtypes. (B) Comparisons of the top 10 altered gene frequency between three subtypes. (C) Comparisons of SETD2 expression between wild and mutated types. (D) K–M plot showing the unfavorable overall survival for patients with low SETD2 expression level. (E) Activated immune‐associated pathways after knockdown SETD2 evaluated based on GSE135105 data set. (F) The ceRNA network analysis for SETD2 downstream miRNAs and genes. (G) Core regulation net of SETD2 regulating PLXNA2 via miR‐497‐5p, miR‐655‐3p, and miR‐374‐5p. (H) The positive correlation between the expression of SETD2 and PLXNA2. ceRNA, competitive endogenous RNA.

For the overall mutation of ccRCC samples in TCGA‐KIRC 225 patients, 86% patients in MoS1, 91% patients in MoS2, and 84% patients in MoS3 contained at least one gene mutation (Figure 3A). MoS2 contained the higher mutation of *PBRM1* and *VHL*, while *SETD2* mutation was mostly observed in MoS1 and MoS3, along with poor prognosis (Figure 3B). For the potential reason of *SETD2* mutation and unfavorable clinical outcome, we first revealed that *SETD2* mutation resulted in the decreased expression *SETD2* mRNA level (*p* < 0.001, Figure 3C), and lower *SETD2* level linked with the shorten OS time (log‐rank test *p* < 0.001, hazard ratio (HR) = 0.56, Figure 3D). In addition, we selected the data from GSE135105, which contains three control samples and three *siDETD2* samples generated from ccRCC cell lines. We observed that siSETD2 activated several immune‐associated signaling pathways, including antigen processing and presentation, cytokine cytokine‐receptor interaction, and natural killer cell‐mediated cytotoxicity (Figure 3E), therefore, less mutation and higher expression of SETD2 might be a reason of lack immunocytes infiltration in MoS2. We constructed the SETD2‐determined competitive endogenous RNA (ceRNA) network (Figure 3F), and revealed that miR‐497‐5p, miR‐655‐3p, and miR‐374‐5p regulated PLXNA2 axis is the hub component (Figure 3G), knockdown SETD2 upregulated the expression of three miRNAs and further inhibited the PLXNA2 level, SETD2 expression positively associated with PLXNA2 expression (*R* = 0.54, *p* < 0.001, Figure 3H). PLXNA2 might be the potential therapy target for SETD2‐mutated ccRCC patients.

### Knockdown of SETD2 facilitated cell proliferation, migration, and invasion of ccRCC cells

**Figure 4:**
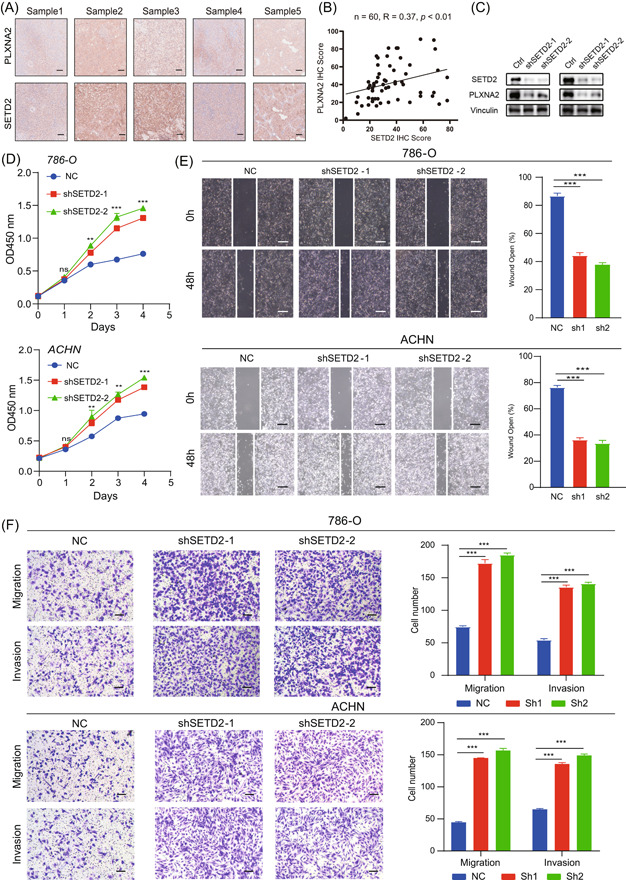
Validation of the function of SETD2 in tumor tissues, 786‐O, and ACHN ccRCC cell lines. (A) SETD2 and PLXNA2 expression was analyzed by immunohistochemistry in 60 ccRCC patients' cancer tissues. Scale bars, 300 μm. (B) Correlation of SETD2 and PLXNA2 IHC score. (C) Knock‐down of SETD2 inhibited PLXNA2 expression. (D) Cell proliferation of 786‐O and ACHN after being transfected with negative control and sh‐SETD2. (E) Wound healing assay of 786‐O and ACHN cells expressing a vector control or sh‐SETD2. Scale bars, 200 μm. (F) Comparison of the number of cell invasion and migration in the SETD2 control and knock‐down of 786‐0 and ACHN cells. Scale bars, 100 μm. ns, not significant; *p < 0.05; **p < 0.01; ***p < 0.001; NC, negative control.

We first collected cancer tissues and assessed the expression of SETD2 and PLXNA2 levels through immunohistochemistry. Through calculating IHC scores of SETD2 and PLXNA2 IHC scores, we found a significant positive correlation of SETD2 and PLXNA2 expression (*n* = 60, *R* = 0.37, *p* < 0.01, Figure 4A,B). In addition, knock‐down of SETD2 could inhibit the expression level of PLXNA2 accordingly (Figure 4C). Those results prove that PLXNA2 might be the downstream target of SETD2.

In addition, CCK8 and wound healing experiment showed that knock‐down of SETD2 facilitated the proliferation and migration capacity of RCC cells (Figure 4D). Consistent with the results above, inhibition of SETD2 also the migration (Figure 4E) and invasion of these cells in the invasion and transwell assays (Figure 4F). Together, these results suggest that SETD2 may function as a tumor suppressor in ccRCC.

### Sunitinib is more suitable for MoS2 and AKT inhibitor more appropriate for MoS1

**Figure 5:**
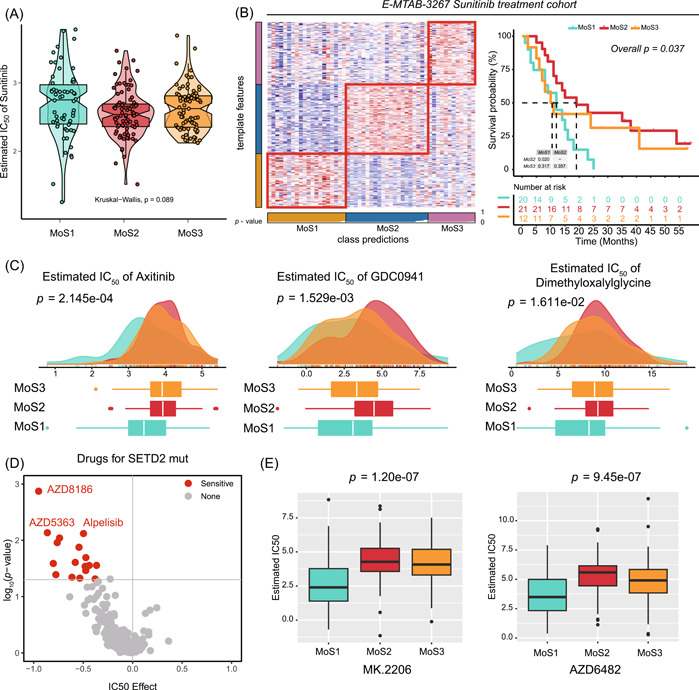
Identify suitable molecular drugs for MoS1 and MoS2 subtypes. (A) Prediction of sunitinib treatment IC_50_ value in TCGA‐KIRC MoS subtypes; (B) clustering of MoS subtypes and Kaplan–Meier plot showing diverse overall survival in E‐MTAB‐3267 sunitinib treated cohort; (C) prediction of axitinib, GDC0941 and dimethyloxalylglycine treatment IC_50_ value in TCGA‐KIRC MoS subtypes; (D) prediction of the potential chemo drugs sensitive to SETD2 mutation; (E) prediction of PI3K/Akt inhibiter treatment IC_50_ value in TCGA‐KIRC MoS subtypes.

To identify the precise chemotherapy for ccRCC patients, we first evaluated the response to sunitinib, patients belonging to MoS2 response more to sunitinib treatment as predicted by the IC_50_ score (*p* = 0.089, Figure 5A). In addition, we recognized the MoSs in E‐MTAB‐3267 cohort, a clinical trial cohort contains 53 ccRCC patients received sunitinib treatment (Table S2). After the treatment of sunitinib, patients belonging to MoS2 met the best OS outcome, MoS1 patients have the worst prognosis (*p* = 0.0037, Figure 5B). As for other usual chemo drugs for ccRCC treatment, we also evaluated and revealed that axitinib, GDC0941, and dimethyloxalylglycine are more suitable for MoS1 patients' treatment (all *p* < 0.05, Figure 5C). We further obtained the sensitive drugs for *SETD2* mutated cells from GDSC online website, we revealed that *SETD2* mutated cells sensitive to inhibitors of PI3K/AKT signaling pathway (AZD8186, AZD5363, Alpelisib, Figure 5D), which was further validated by the PI3K/AKT inhibitors of MK.2206 (*p* < 0.001) and AZD6482 (*p* < 0.001) (Figure 5E).

### Reproduced the MoSs and validated the diverse clinical outcomes in external cohorts

**Figure 6:**
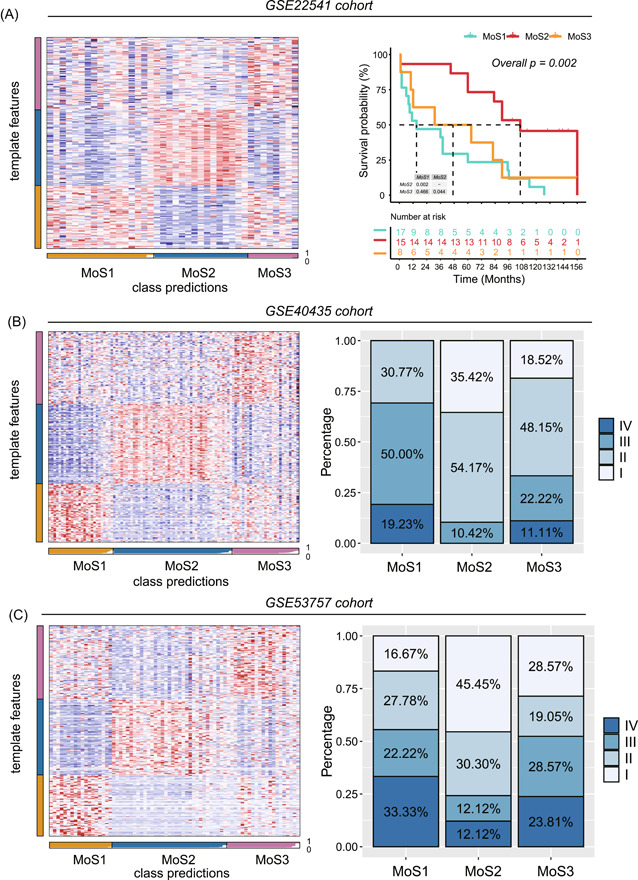
External validation of MoS classification by subtype‐specific markers. (A) Representing the MoS classification in GSE22541 cohort and assessing the diverse overall survival by Kaplan–Meier plot; (B) representing the MoS classification in GSE22541 cohort and analyzing the distribution of tumor stage; (C) Representing the MoS classification in GSE53757 cohort and analyzing the distribution of tumor stage. MoS, multiomics subtype.

With the 300 subtype‐specific markers, we reproduced the MoSs in external cohorts (Table S2). Forty patients from GSE22541 seperated to MoS1, MoS2, MoS3, and patients belonging to MoS2 met the best prognosis, while MoS1 patients' clinical outcome is the worst (Figure 6A). Further multivariate Cox regression analysis adjusted the bias of gender, and MoSs is still the independent prognostic factor for ccRCC (Table 2). As for GSE40435, patients in MoS2 were mostly at the early tumor stage (Stage I, 10.42; Stage II 54.17), which might indicate favorable prognosis, and MoS1 contained the most patients at Stage IV (19.23%) (Figure 6B). Results in GSE53757 displayed the similar tendency to support the above findings, about 50% patients belonging to MoS2 at early Stage I, while 33.33% patients were belonging to MoS1 at Stage IV (Figure 6C). We also further validated the molecular features of MoS subtypes in external cohorts, we observed that MoS1 and MoS3 contained the high infiltration of immunocytes, MoS1 also contained the dramatical activation of immune exhausted signatures, such as TITR, C‐ECM, while MoS2 is immune‐cold subtype (Figure S3). For the signaling pathways, MoS1 patients contained the activation of extracellular structure, collagen metabolism, and acute inflammatory response associated pathways; MoS2 patients met the enrichment of fatty acid metabolic and catabolic, monocarboxylic acid catabolic; the activation of immune‐associated pathways enriched in MoS3 significantly, including immune cell differentiation, antigen processing and presentation (Figure S3).

**Table 2:** Newly defined cancer subtypes are independent prognostic factors for ccRCC patients.

Parameters	HR	95% CI	*p* Value
TCGA−KIRC cohort			
Age	1.058	1.027−1.089	<0.001*
Gender			
Female	Ref.	−	−
Male	0.737	0.417−1.300	0.291
Grade			
G1 + G2	Ref.	−	−
G3	0.768	0.365−1.619	0.488
G4	1.537	0.647−3.652	0.33
Stage			
I	Ref.	−	−
II	1.992	0.689−5.756	0.203
III	2.194	0.986−4.882	0.054
IV	4.761	2.184−10.383	<0.001*
Subgroup			
MoS2	Ref.	−	−
MoS1	5.106	1.960−13.303	0.001*
MoS3	4.173	1.722−10.113	0.002*
GSE25541 cohort			
Gender			
Female	Ref.	−	−
Male	0.552	0.220−1.390	0.207
Subgroup			
MoS2	Ref.	−	−
MoS1	5.975	2.044−17.470	0.001*
MoS3	3.969	1.266−12.448	0.018*

Abbreviations: CI, confidence interval; OR, odds ratio.

* *p* < 0.05.

## DISCUSSION

Despite ccRCC being generally considered as an early‐detectable disease that can be cured via ablative treatment or resection policies, one‐third of patients might suffer from metastases, and develop to a succumb disease [52]. Similar to other tumors, the heterogeneity of ccRCC prognosis stems from intrinsic molecular alterations. Currently, the development of high‐throughput sequencing and bioinformatics promotes the elucidation of comprehensive molecular alteration landscape in ccRCC. Many novel risk stratification schemas were established based on different altered molecules and forms. For instance, TRACERs renal program assigned ccRCC into seven major subtypes, including VHL monodriver, PBRM1‐SETD2, PBRM1‐somatic copy number alteration (SCNA), PBRM1‐PI3K, VHL wildtype, multiple clonal driver, and BAP1 driven, these genomically distinct subtypes exhibited significantly heterogeneous prognosis [53]; Peng and colleagues developed a methylation signature for predicting survival for ccRCC, and the high‐methylation score group exhibited longer OS than low‐methylation score group (HR = 2.46, 95% confidence interval (CI): 1.63–3.71, *p* < 0.001) [54]. On the other side of the spectrum, molecular alterations at different cellular levels in ccRCC might hinder the identification of reliable risk stratification schemes, therefore, we must reveal molecular subtypes based on multiple perspectives against the limitations caused by only one certain omics in present studies.

**Figure 7:**
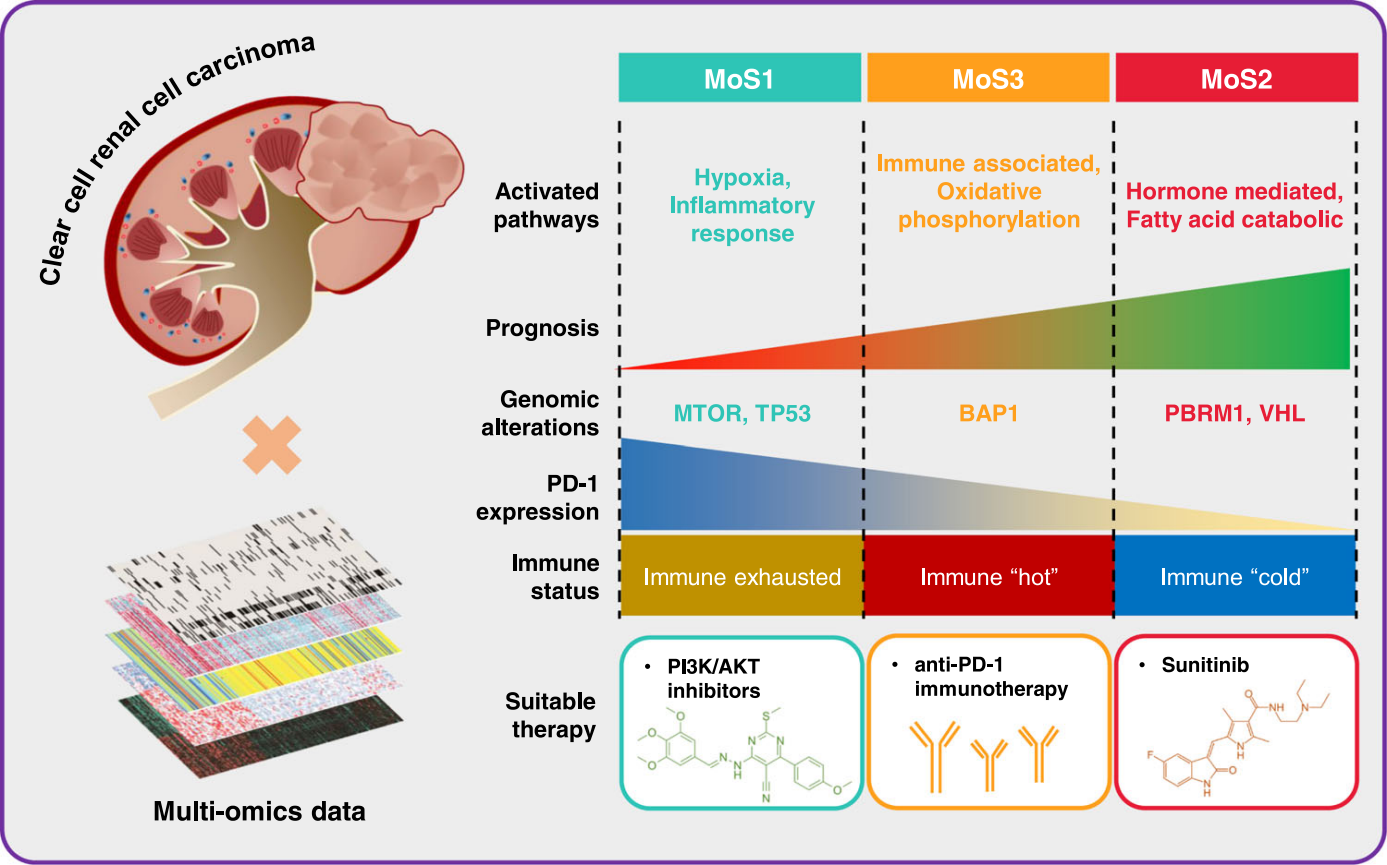
Summary characteristics of the MoS classification. Sketch diagram shows the activated pathways, genetic alterations, immune status, PD‐1 expression, immune status, and the suitable therapy among three MoS classifications. MoS, multiomics subtype.

Using 10 clustering algorithms applied to investigate the correlation between integrative data and OS outcomes, we established a consensus classification, which fully considered the heterogeneity of multiple omics of ccRCC cells, including the features of mRNA, lncRNA expression, CNAs, DNA methylation, and gene mutation. MoS1 subtype presented the worst prognosis, MoS2 subtype was the prognostic best phenotype and MoS3 subtype was moderate. In addition, these three distinct phenotypes showed significantly different molecular alteration landscape and signaling pathways activation, thereby leading to variable metabolic processes and biological behaviors (Figure 7).

Malignant tumors are characterized by cycling hypoxia as their rapid growth corresponds to unmatched development of blood vessels, and hypoxia conditions would increase the growth rate, promote migration and metastases of tumors in return [55]. Compared to MoS1 and MoS3, MoS2 phenotypes harbored lower hypoxia scores, strongly suggesting MoS2 represent a relatively indolent and low invasive potential subtype. VHL mutation is the hallmark oncogene in ccRCC. The correlations between VHL mutation and ccRCC prognosis are controversial; positive, negative, and irrelevant are proven. A meta‐analysis included five VHL mutation‐relevant studies revealed no correlations between mutated VHL and prognosis of ccRCC but have protective trends [56]. In the present study, more VHL mutation was enriched into MoS2 subtype, which is linked to a longer OS than other phenotypes, suggesting protective trends as well. MoS1 and MoS3 phenotypes exhibited high DNA methylation burden. DNA methylation modify genetic information and impact expression without changing the DNA sequence, hypermethylation of enhancer and promoter CpGs contribute to epigenetic silence of abundant of tumor suppressor genes, thereby promoting proliferation, invasion, and metastasis of ccRCC [57]. CNA involves in chromosomal losses or gains, which can impact multiple genes, and with the dosage accumulation, the expression level of relevant genes would be altered as the consequence [58]. Our results demonstrated that the deletion of chromosome 9 is another diverse event in ccRCC, linked to an advanced stage and a poor clinical outcome. This finding is consistent with several prior studies, which might be caused by the deficiency of tumor suppressor gene, CDKN2A located on 9p21 [59], CAIX located on 9p13 [60], and NDUFB6 located on 9p24.1‐p13.3 [61].

SETD2 located in chromosome 3p, encodes the expression of The H3 lysine 36 histone methyltransferase and was first reported in ccRCC [62]. SETD2 deficiency would promote tumors development through various mechanisms, such as inhibition of DNA repair, change cellular mechanism, activation of oncogene genes, and perturbation of cell cycle progression [63, 64, 65, 66]. Serving as a protective factor in ccRCC, mutated SETD2 was reported in 34.07% of patients and linked to the invasion phenotypes [67]. In the present study, high mutations of SETD2 were observed among MoS1 and MoS3 subtypes, which was consistent with their poor clinical presentation. These evidence indicated the pivotal role of SETD2 promote ccRCC progression, and we therefore performed further studies. Based on ceRNA network analysis, we revealed the downstream key gene, PLXNA2, which can be regulated by SETD2 via miR‐497‐5p, miR‐655‐3p, and miR‐374‐5p. IHC validated the positive correlation between SETD2 and PLXNA2, and in the STED2 knock‐down 786‐O and ACHN cell lines, ccRCC presented significantly decreased expression of PLXNA2, proving the result from ceRNA network. Meanwhile, STED2 knock‐down ccRCC cells exhibited high tendency of proliferation, migration, and invasion, reconfirming SETD2 function as a tumor suppressor in ccRCC.

We further investigated the immunocytes infiltration status and expression level of PD‐1 in three MoS subtypes. The highest expression level of PD‐1 and immune signatures were found in MoS1, but accompanied with the activation of TITR and C‐ECM signatures, indicating an immunogenically “cold” phenotype and therefore should conceptually be low response to PD‐1 blockade. Meanwhile, patients in MoS3 subtype would benefit more from anti‐PD‐1 therapy in this study as compared with that in MoS1 subtype despite relatively less activation of immune suppress pathways. In line with our expectation, the expression of PD‐1 is one of the aspects that determine the efficacy of anti‐PD‐1 therapy, and immune‐activated status also plays a hub role [41]. C‐ECM, TITR signatures were manifested as marks of exhausted immune status, and the high exhausted score of MoS1 indicated a poor response to anti‐PD‐1 therapy. In contrast, high infiltration of immunocytes in MoS3 ccRCC cells implied an activated immune status and a high response to anti‐PD‐1 therapy. Particularly, different types of macrophage infiltration may be one of the reasons for the different prognosis among MoS subtypes, M1 macrophage was highly enriched in MoS2 phenotype, and was considered to positively regulate the immune response, thereby killing tumor cells, while MoS3 ccRCC harbored high M2 macrophages infiltration, which secrete kinds of growth factors and remodel tumor microenvironment to support tumor progression, invasion and metastasis [68].

Local advanced or metastatic disease account for nearly one‐third of all ccRCC cases, and have poor limited responsiveness to typically adjuvant therapies, such as cytokine therapy, chemotherapy, or radiotherapy [69]. The highly vascularized features of ccRCC provided a chance for antiangiogenic therapy, and sunitinib have been used as the standard first‐line treatment for metastatic ccRCC [70]. However, more than half of patients cannot benefit from sunitinib, thus, identifying the appropriate patients will improve the efficacy of sunitinib. In this study, MoS2 patients exhibited high‐sensitive to sunitinib, while MoS1 showed poor responsiveness. For patients in MoS1 subtype, axitinib, GDC0941, and dimethyloxalylglycine are more likely to delay progression of ccRCC. PI3K‐AKT pathway was widely reported as a promising druggable target in SETD2‐deficient ccRCC [71], indicating the inhibitors of PI3K‐AKT pathway might be suitable for MoS1 and MoS2 subtypes for their high SETD2 mutations, and based on GDSC online website, we identified several drugs for MoS1 as well, such as AZD8186, AZD5653, and Alpelisib. Collectively, the results mentioned above suggested MoSs classifier might be a potential tool for developing individualized treatment strategies for different patients.

## CONCLUSION

Taken together, we established a novel molecular classification for ccRCC via multiomics features. MoS subtypes correlate to significantly different clinicopathological parameters, molecular alterations, signaling activation, immunocyte infiltration, and therapeutic response. MoS1 subtype, presented the poorest prognosis, might be caused by an exhausted immune microenvironment, activated hypoxia features, but can benefit from PI3K/AKT inhibitors. MoS2 subtype represented more mutation of VHL and PBRM1, favorable prognosis, and is more suitable for sunitinib therapy. MoS3 is the immune “hot” subtype, and can benefit from the anti‐PD‐1 immunotherapy. We also confirm that SETD2 is a tumor suppressor in ccRCC, along with the decreased SETD2 protein level in advanced tumor stage, and knock‐down SETD2 lead to the promotion of cell proliferation, migration, and invasion. To further test MoS classification, a longitudinal and prospective study is urgent to provide more evidence.

## METHODS

### Multiple omics data enrolled

TCGA‐KIRC cohort was enrolled for the multiomics analysis, which included complete transcriptome expression profiles, survival outcomes, copy number alterations, DNA methylation, and somatic mutations data. We used the R package “TCGAbiolinks” to download the row counts of mRNA and lncRNA expression profiles [30]. LncRNA data were recorded as noncoding, 3prime overlapping ncRNA, antisense RNA, lincRNA, sense intronic, sense overlapping, macro lncRNA, and bidirectional promoter lncRNA subtypes and were identified according to Vega (http://vega.archive.ensembl.org/). Ensembl IDs for these transcriptomes were converted into gene symbols by GENCODE27 mapping. We obtained Illumina DNA methylation data (https://xenabrowser.net/). miRNA data was dropped due to the lack of date in large number of TCGA‐KIRC patients. Furthermore, somatic mutations, clinicopathological features were retrieved from cBioPortal (https://www.cbioportal.org/). To obtain the training cohort, we filtered the patients, and only enrolled the ccRCC patients with five omics data and OS outcome, finally, a total of 225 patients left for the subsequent analysis. To validate the results of the training cohort, we collected seven microarray data sets. GSE22541, GSE40435, and GSE53573 cohorts were enrolled to verify the diverse clinical outcomes of molecular subtypes, with the available clinical data of OS outcome, tumor stage, or tumor grade. Forty‐five patients from CheckMate cohort [31] and 16 patients from Miao cohort [32] who received Nivolumab (anti‐PD‐1) were enrolled to evaluate their sensitivity to immunotherapy. Fifty‐three patients who received sunitinib treatment from E‐MTAB‐3267 cohort were enrolled to reveal the effectiveness of sunitinib treatment. Based on the annotation files from the corresponding platform, the probe ID for each enrolled cohort was transferred to the gene symbol. If the gene symbol was annotated with multiple probe IDs, the median value was considered. All of the clinicopathological features of the enrolled cohorts are listed in Tables 3 and S3.

**Table 3:** Basic clinical features of the enrolled cohorts.

Characteristics	TCGA−KIRC	GSE22541	GSE40435	GSE53757	EMTAB3267	CheckMate	Miao
Number of samples	225	40	101	72	53	45	16
Treatment	**−**	**−**	**−**	**−**	Sunitinib	Nivolumab	Nivolumab
Gender							
Male	146	24	59	**−**	37	30	13
Female	79	16	42	**−**	16	15	3
Age, years							
Mean ± SD	60.98 ± 11.41	**−**	64.12 ± 9.24	**−**	59.85 ± 8.22	61.80 ± 9.23	62.88 ± 6.82
Grade							
G1	4		22	**−**	**−**	**−**	**−**
G2	90		47	**−**	**−**	**−**	**−**
G3	91		24	**−**	**−**	**−**	**−**
G4	37		8	**−**	**−**	**−**	**−**
Unknown	3						
Stage							
I	112	**−**	**−**	24	**−**	**−**	**−**
II	24	**−**	**−**	19	**−**	**−**	**−**
III	50	**−**	**−**	14	**−**	**−**	**−**
IV	37	**−**	**−**	15	**−**	**−**	**−**
Unknown	2						
Immunotherapy							
Nonclinical benefit	**−**	**−**	**−**	**−**	**−**	20	8
Clinical benefit	**−**	**−**	**−**	**−**	**−**	25	8

### Raw data integration and classification of molecular subtypes

We established a novel classification of ccRCC based on the multiomics data of mRNA expression, lncRNA expression, miRNA expression, DNA methylation, CNAs, and somatic mutation data. To select the needed information, these enrolled data were first processed. We performed log_2_ (TPM+ 1) calculation on the transcriptome data to make it more comparable. For the DNA methylation data, we only concerned the probes at which located promoter region CpG island, and for genes having more than one probe mapping to its promoter, the median *β* value was considered. As to gene mutation, the samples with nonsynonymous variations in the gene mutation matrix were retained for the subsequent analysis, which included frameshift deletion/insertion, in‐frame deletion/insertion, missense/nonsense/nonstop mutation, and splice site or translation start site mutation. For the CNAs, we condensed the genomic segments as described in the literature [33]. We removed the features from which the values were flat to accelerate clustering efficiency. To better match the subtypes with their corresponding clinical information, we extracted the factors that were most relevant to the OS according to the Cox regression survival analysis, these factors that with the *p* < 0.001 were considered, and finally enrolled 664 mRNAs (Table S4), 60 lncRNAs (Table S5), 60 CNAs (Table S6), 569 DNA methylation sites (Table S7). 14 genes with mutant frequency higher than 5% were also enrolled for multiomics analysis. To find the most optimal assembling numbers, CPI and Gap statistics were calculated as well as referred to the previously reported RCC molecular subtypes [22, 34, 35, 36]. Subsequently, 10 state‐of‐the‐art‐multiomics were performed to establish the final classification model, including iClusterBayes, moCluster, CIMLR, IntNMF, ConsensusClustering, COCA, NEMO, PINSPlus, SNF, and LRA as described in the R package “MOVICS” which we developed recent days [37]. Further integration of the clustering results from the above‐mentioned algorithms was applied to generate robust clustering.

For the seven external cohorts, the neatest template predictions (NTP) were conducted to separate the expression data into different clusters referenced by the biomarkers generated from the training TCGA‐KIRC cohort [38]. The most differentially expressed genes sorted by log_2_FoldChange are chosen as the biomarkers for each subtype, these biomarkers should pass the significance threshold (adjusted *p* < 0.05) and must not overlap with any biomarkers identified for other subtypes.

### Evaluation of diverse molecular features

For all the newly defined molecular subtypes, it is critical to depict the characteristics validated by different signatures of gene sets. To reveal the latent biological characteristics, GO terms were used to characterize the subtypes by gene set variation analysis (GSVA). The identified subtype‐specific pathways should meet the significance threshold, with the adjusted *p* < 0.25, and any pathways overlapped with other subtypes would be removed. The top 10 GO pathways and the relationship networks among subtypes were visualized.

As for the immunocytes abundance, we assessed it to each patient with the CIBERSORT algorithm [39], and the average infiltration rate of patients in each subtype was visualized by heatmap to indicate the different immune landscape. We also evaluated the immune‐associated signatures which were used in our prior study, to further distinguish the immune‐activated and immune‐exhausted features [40, 41]. The enrichment scores for each signature were calculated by the R package “GSVA,” and further displayed with the R package “ComplexHeatmap” [42].

For the CNA and gene mutations in different subtypes, the individual fraction of copy number‐altered genome (FGA) for the MIBC‐TCGA cohort was calculated based on copy number segment data as follows:

R=copynumberofsegments/2

$$\mathrm{FGA}=B_{r}/B$$

The FGA is a fraction of the genome with a log_2_(copy number) value larger than 0.3 versus the genome with a copy number profiled where $B_{r}$ denotes the number of bases in segments with $|\log_{2}R|>0.3$ and B represents the number of bases in all segments. The alteration event, including CNA and gene mutation, among different subtypes was displayed with the cBioProtal, as well as the gained or lost CNA‐specific chromosome. Winter hypoxia score, buffa hypoxia score, and ragnum hypoxia score were also downloaded from the cBioProtal. The alteration event frequency among wild‐type and mutant SETD2 samples was compared by the Wilcoxon test.

### Prediction of precise therapy strategies

We evaluated the curative effect of patients among clusters of chemotherapy, including routine ccRCC chemotherapy, anti‐hypoxia therapy, and PI3K/Akt pathway inhibitors. To estimate the chemotherapeutic responses of each individual, 727 human cancer cell lines (CCLs) from the GDSC (https://www.cancerrxgene.org/) were used as the training cohort and the R package “pRRohetic” was employed to predict the corresponding sensitivity [43]. Half maximal inhibitory concentration (IC_50_) was calculated by ridge regression and set as an index to compare different agents. The therapeutic responses of two common chemotherapeutic agents (sorafenib and sunitinib), three antihypoxia agents (Axitinib, GDC0941, and Dimethyloxalylglycine), and three PI3K/Akt pathway inhibitors (A.443654, MK.2206, and AZD6482) are quantified by IC_50_, respectively. Lower IC_50_ indicated increased sensitivity to treatment, and 10‐fold cross‐validation was adopted to measure the prediction efficacy. As mentioned above, 53 patients which received sunitinib treatment from E‐MTAB‐3267 cohort were enrolled to reveal the effectiveness of sunitinib treatment.

We are also concerned about immunotherapy by anti‐PD‐1. 47 melanoma patients receiving anti‐CTLA‐4 or anti‐PD‐1 checkpoint inhibition therapy with 795 immune‐related gene profile were included. Subclass mapping analysis (GenePattern module “SubMap”) [44], which reveals common subtypes in independent datasets, was applied to detect similarity of gene expression profiles between our subtypes. What's more, we also enrolled 45 patients from CheckMate cohort and 16 patients from Miao cohort who received Nivolumab (anti‐PD‐1) were enrolled to evaluate their sensitivity to immunotherapy.

### Western blot and immunohistochemistry

Cells were collected and lysed with RIPA buffer containing phenylmethanesulfonylfluoride (Beyotime). Protein concentration was determined with a BCA assay kit (Beyotime). The primary antibody information used is as follows: anti‐SETD2 (1:1000, #80290; Cell Signaling), anti‐PLXNA2 (1:1000, #DF9760; Affinity) and anti‐Vinculin (1:5000, 66305‐1‐Ig; Proteintech). The paraffin sections of ccRCC tissues were obtained from the ccRCC patients that received surgery in the urology department of Shanghai Changhai Hospital, and used to perform immunohistochemical staining to measure the protein levels of SETD2 (1:200; PA5‐34934; Thermo Fisher Scientific) and PLXNA2 (1:500, ab39357; Abcam), respectively.

### Investigation of SETD2 biological function in vitro

Human ccRCC cell lines 786‐O and ACHN were purchased form the Chinese Academy of Science. ACHN cell was maintained in MEM (Gibco). 786‐O cell was maintained in RPMI‐1640 medium (Gibco). All culture mediums of cell lines were supplemented with fetal bovine serum (FBS, 10% Gibco) and 1% penicillin/streptomycin (Gibco). All cell lines were cultured at 37°C and 5% CO_2_ within 40 generations. All cell lines in this study were identified through short tandem repeat (STR) analysis and examined for mycoplasma contamination using a mycoplasma test kit (Selleck Chemicals). We applied 4′,6‐diamidino‐2‐phenylindole (DAPI) staining to test whether cell lines infected with mycoplasma. All cell lines were cultured according to standard protocol. SETD2 knock‐down plasmid and negative control were constructed by GeneChem. The SETD2 knock‐down and the negative control plasmids were transfected into the 293T cells by using Lipofectamine 2000 (Invitrogen; Thermo Fisher Scientific, Inc.) to package the lentiviruses (Table S8). 786‐O and ACHN cells were cultured in six‐well culture dishes at 60% density and then infected with SETD2 knock‐out lentivirus and the negative control lentivirus. All the transfections were supplied with 4 µg/mL Polybrene (H8761; Solarbio, Inc.) and lasted for 12 h. Screening was conducted with 2 µg/mL puromycin (P8833; Sigma, Inc.) for 3 days to acquire stably transfected cells.

### Cell proliferation assay

RCC cell lines were seeded in 96‐well plate at a density of 2 × 10^3^ cells/100 µL per well and incubated overnight. After density of cell lines reached to 70%, CCK8 reagent (Dojin Laboratories) was added and measured at 0, 24, 48, 72, and 96 h. Absorbance was measured at a wavelength of 450 nm with reference wavelength of 570 nm.

### Cell wound‐healing assay

In this part, 786‐O and ACHN cells were seeded into a six‐well plate and incubated in medium containing 10% FBS overnight. The wound was scraped using a 200 μL pipette tip and washed with PBS to remove cell debris. Finally, the supernatant was replaced with a serum‐free medium. Scratch gaps were photographed at 0 and 24 h using a microscope (Olympus).

### Invasion and transwell assay

Transwell chambers (Corning) were prepared with or without Matrigel. The metastatic abilities of ccRCC cell lines were calculated through invasion and migration assay. 786‐O and ACHN cells were inoculated into a six‐well plate for 48 h. Cells were harvested with 0.25% trypsin and resuspended in serum‐free medium. For the cell invasion assays, 4 × 10^4^ cells/200 μL per well were seeded in a transwell chamber pretreated coated with Matrigel in the upper chamber. Five hundred microliters complete medium (containing 10% FBS) was placed in the lower chamber as an inducer. After 24 h, a cotton swab was used to remove cells located in the upper chamber; cells on the lower side of the chamber were fixed with 4% paraformaldehyde (Beyotime) and stained with 0.5% crystal violet solution. Finally, the cells were counted and photographed using a microscope (Olympus).

### Statistical analyses

All the statistical analyses were performed with R version 4.0.2 (https://www.r-project.org/). For the continuous variables, Student's *t* test and a two‐sample Mann–Whitney were used to compare two groups if the data was normally distributed, or Wilson rank test was applied. Before the comparisons of the normality of the distributions, Shapiro–Wilk test would be conducted. For categorical variables, Chi‐square test and Fisher's exact test would be performed. For the comparison of gene mutation frequencies among clusters, Permutation test was performed. Relying on the log‐rank test, Kaplan–Meier was generated to analyze the OS. Cox regression model was established to calculate the HR values and the 95% CI. Most of the analyses above were performed based on the R package “MOVICS” [37]. To evaluate the biological differences among clusters, GSEA was performed with the R package “clusterProfiler” based a prerank gen list which was ordered by log_2_FoldChange algorithms from the “limma” package [45, 46]. Pearson correlation test was conducted to calculate the coefficient values and *p* < 0.05 was considered to be statistically significant.

## AUTHOR CONTRIBUTIONS

Jialin Meng conducted the conceptualization, designed the methodology, performed formal analysis, wrote the original draft, reviewed and edited the manuscript. Aimin Jiang validated the results, contributed resources, and wrote the original draft. Xiaofan Lu conceptualized the study, designed the methodology, and provided resources. Di Gu validated the results, provided resources, and carried out the investigation. Qintao Ge performed formal analysis, validated the results. Suwen Bai validated results, wrote the original draft, and contributed to the investigation. Yundong Zhou contributed to the methodology, performed formal analysis. Jun Zhou provided resources, validated results, and wrote the original draft. Zongyao Hao conceptualized the study, provided resources, and curated data. Fangrong Yan designed the methodology and supervised this project. Linhui Wang provided resources, and reviewed and edited the manuscript. Haitao Wang conceptualized the study and reviewed & edited the manuscript. Juan Du conceptualized the study, reviewed and edited the manuscript, and supervised the project. Chaozhao Liang conceptualized the study, provided resources, reviewed & edited the manuscript, supervised the project, and provided funding. All authors have read the final manuscript and approved it for publication.

## CONFLICT OF INTEREST STATEMENT

The authors declare no conflict of interest.

## ETHICS STATEMENT

The procedure related to human subjects was approved by the Ethics Committee of the Changhai Hospital (No. CHEC2021091). Consents from participants were waived, because the current study was based on the existing pathology specimens, and will not adversely affect the rights and welfare of the subjects. As the other data used in this study are publicly available, no ethical approval was required.

## Supporting information

Supporting information.

Supporting information.

## Data Availability

Public data used in this study are available in TCGA, ArrayExpress, and GEO. The analytic processes in this study are embedded in the R package “MOVICS” at https://github.com/xlucpu/MOVICS, which we recently developed for multiomics integration and visualization. The code and data of this study is available in Github (https://github.com/AHMUJia/ccRCC_MOS). Supplementary materials (figures, tables, scripts, graphical abstract, slides, videos, Chinese translated version and update materials) may be found in the online DOI or iMeta Science http://www.imeta.science/.
