# Supplementary material for: Multiomics characterization and verification of clear cell renal cell carcinoma molecular subtypes to guide precise chemotherapy and immunotherapy
Source: Imeta. 2023 Nov 16;2(4):e147. doi: 10.1002/imt2.147 (PMC10989995; doi:10.1002/imt2.147)
Supplement: Supplementary file 1 — Supporting information. [file IMT2-2-e147-s002.docx]

**Supporting information to**

**Multi-omics characterization and verification of clear cell renal cell carcinoma molecular subtypes to guide precise chemotherapy and immunotherapy**

**Running title:** Multi-Omics Reveal Subtypes for Clear Cell Renal Cell Carcinoma

Jialin Meng^1,#,*^, Aimin Jiang^2,#^, Xiaofan Lu^3,#^, Di Gu^2^, Qintao Ge^1^, Suwen Bai^4^, Yundong Zhou^5^, Jun Zhou^1^, Zongyao Hao^1^, Fangrong Yan^6^, Linhui Wang^2^, Haitao Wang^7, †^, Juan Du^4,*^, Chaozhao Liang^1,*^

^1^ Department of Urology, The First Affiliated Hospital of Anhui Medical University; Institute of Urology, Anhui Medical University; Anhui Province Key Laboratory of Genitourinary Diseases, Anhui Medical University, Hefei 230022, China

^2^ Department of Urology, Changhai Hospital, Naval Medical University (Second Military Medical University), Shanghai 200433, China

^3^ Department of Cancer and Functional Genomics, Institute of Genetics and Molecular and Cellular Biology, CNRS/INSERM/UNISTRA, 67400 Illkirch, France.

^4^ The Second Affiliated Hospital, School of Medicine, The Chinese University of Hong Kong, Shenzhen & Longgang District People’s Hospital of Shenzhen, Shenzhen 518172, China

^5^ Department of Surgery, Ningbo Medical Center Lihuili Hospital, Ningbo University, Ningbo, Zhejiang 315040, China

^6^ Research Center of Biostatistics and Computational Pharmacy, China Pharmaceutical University, Nanjing 211198, China.

^7^ Cancer Center, Faculty of Health Sciences, University of Macau, Macau SAR, China.

^†^ Current address: Center for Cancer Research, Clinical Research/NCI/NIH, Bethesda, MD 20892, USA

# These authors contributed equally to the study.

*Correspondence:

**Jialin Meng,** Department of Urology, The First Affiliated Hospital of Anhui Medical University; Institute of Urology, Anhui Medical University; Anhui Province Key Laboratory of Genitourinary Diseases, Anhui Medical University, Hefei 230022, China

Email: [mengjialin@ahmu.edu.cn](mailto:mengjialin@ahmu.edu.cn)

**Juan Du,** School of Medicine, The Chinese University of Hong Kong, Shenzhen; The Second Affiliated Hospital，School of Medicine, The Chinese University of Hong Kong, Shenzhen, 518172, Guangdong, China.

Email: [dujuan@cuhk.edu.cn](mailto:dujuan@cuhk.edu.cn)

**Chaozhao Liang**, Department of Urology, The First Affiliated Hospital of Anhui Medical University; Institute of Urology, Anhui Medical University; Anhui Province Key Laboratory of Genitourinary Diseases, Anhui Medical University, Hefei 230022, China

Email: [liang_chaozhao@ahmu.edu.cn](mailto:liang_chaozhao@ahmu.edu.cn)

**
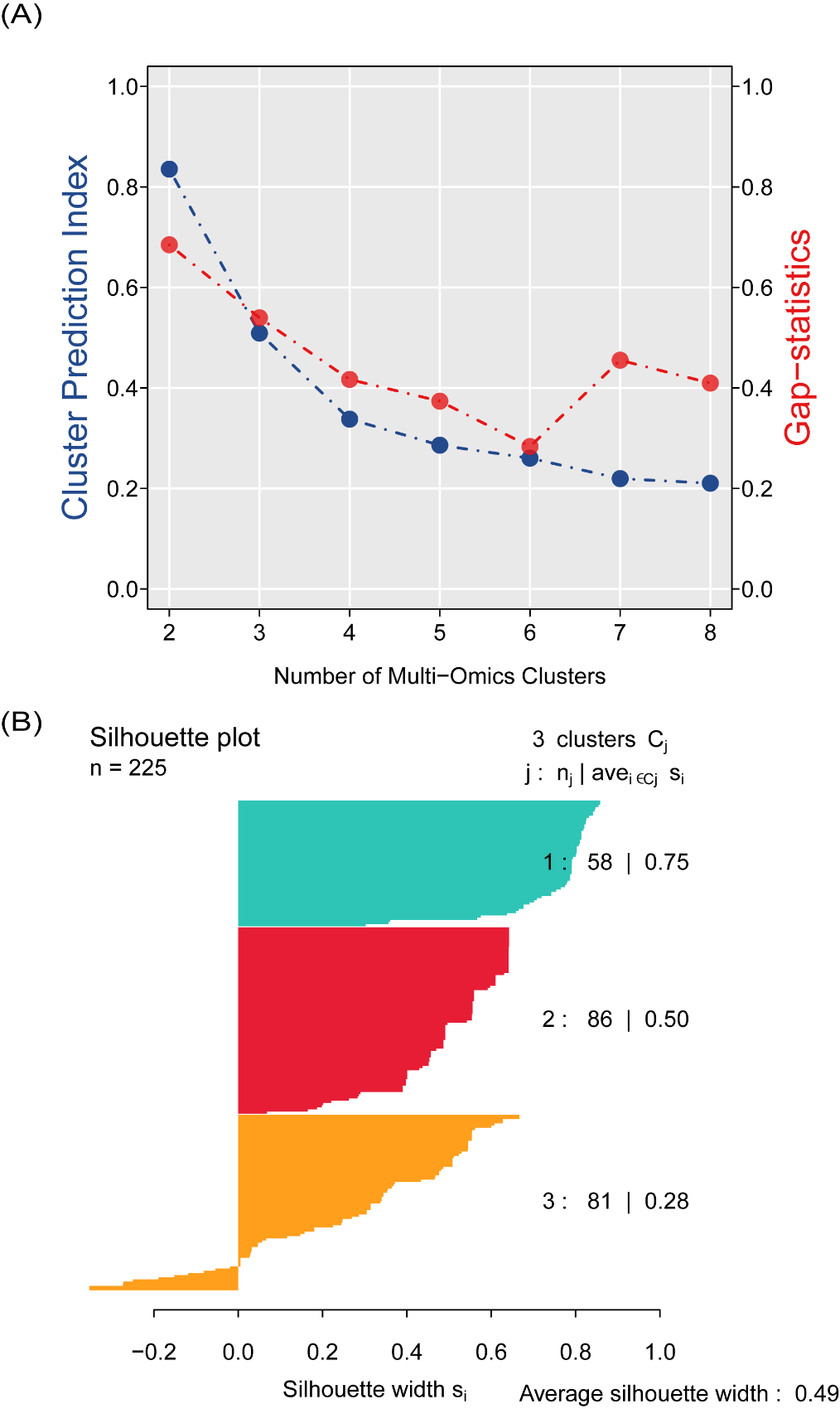
**

**Figure S1. Identify molecular subtypes through multi-omics data integration.** (A) Determination of the optimal number of multi-omics clusters using the cluster prediction index and Gap-statistics. (B) Evaluation of sample homogeneity through silhouette scores derived from consensus ensemble results.

**
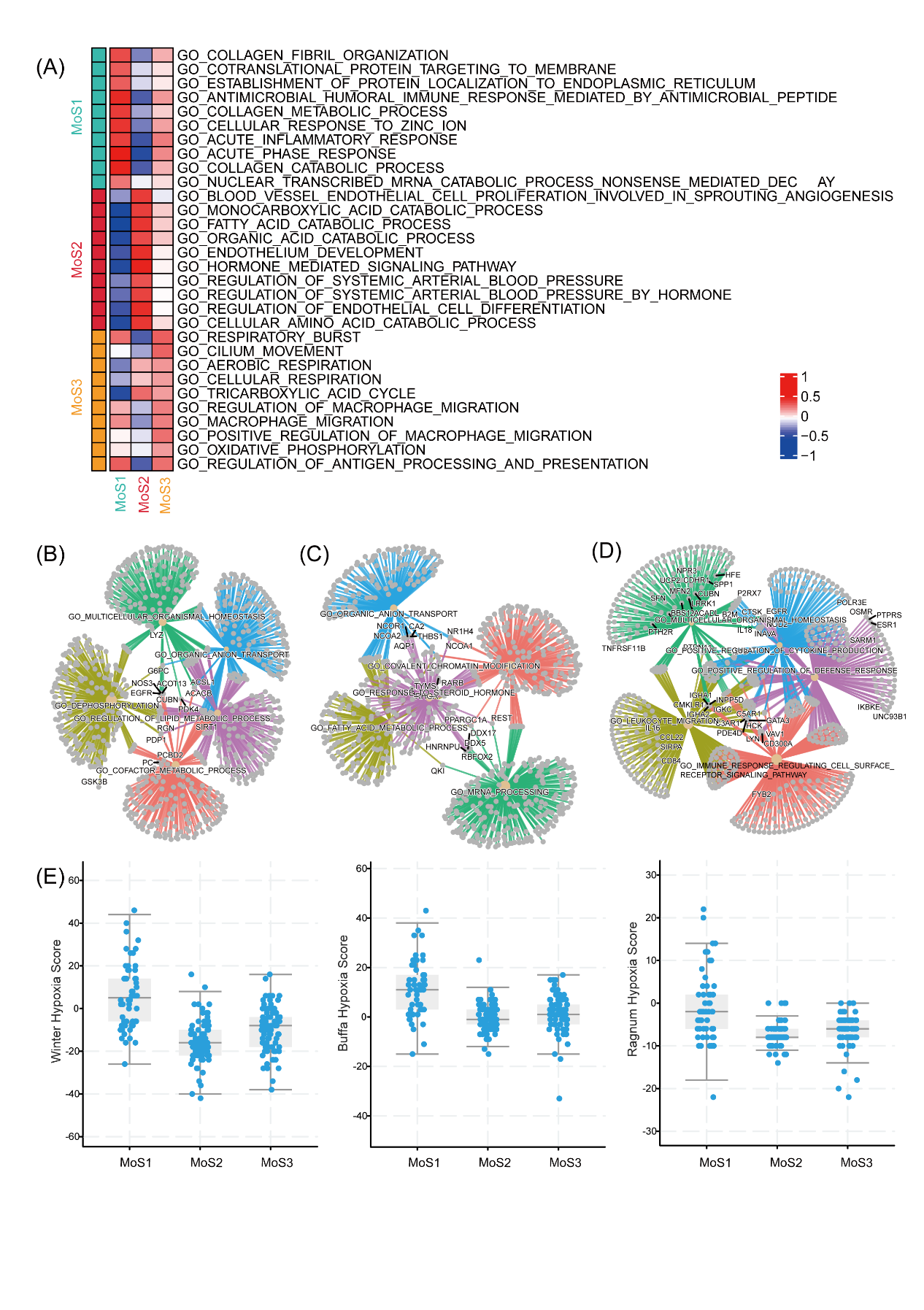
Figure S2. Biological pathways enrichment and hypoxia score comparison.** (A) Top ten enriched biological pathways among MoS1, MoS2 and MoS3 subtypes. (B) Pathway enrichment network for MoS1 subtype. (C) Pathway enrichment network for MoS2 subtype. (D) Pathway enrichment network analysis for MoS3 subtype. (E) Comparisons of hypoxia score among three MoSs. MoS, multi-omics subtype.


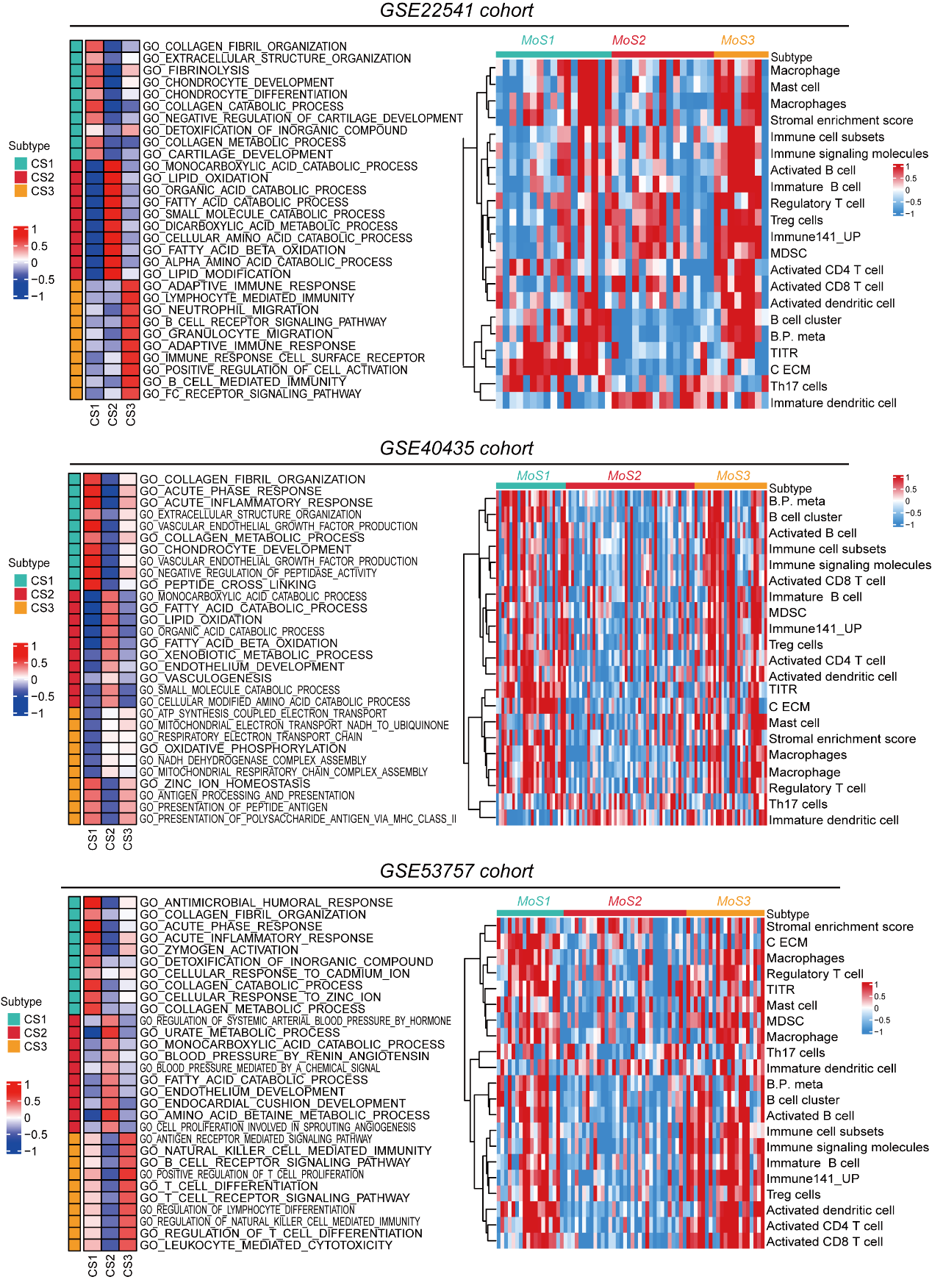


**Figure S3. External validation of molecular features in different subtypes.** Evaluation of specific pathway enrichment and immune cell infiltration in GSE22541, GSE40435, and GSE53757 cohorts. TITR, tumor-infiltrating T regulatory cell; C-ECM, cancer-associated extracellular matrix; MDSC, Myeloid-derived suppressor cell.
